# Effectiveness of Low-Level Laser Therapy Combined With Eccentric Exercise in Treating Midportion Achilles Tendinopathy: A Randomized Controlled Trial

**DOI:** 10.7759/cureus.62919

**Published:** 2024-06-22

**Authors:** Surbhi Shriya, Rajendra K Arya, Sushmita Kushwaha, Soni Chahar, Manikandan P, Pankaj Mehra

**Affiliations:** 1 Sports Medicine, Sports Injury Centre, Vardhman Mahavir Medical College (VMMC) & Safdarjung Hospital, New Delhi, IND; 2 Orthopedics, Atal Bihari Vajpayee Institute of Medical Sciences (ABVIMS) & Dr. Ram Manohar Lohia (RML) Hospital, New Delhi, IND; 3 Sports Medicine, Pandit Bhagwat Dayal Sharma Post Graduate Institute of Medical Sciences, Rohtak, IND

**Keywords:** achilles tendon, overuse injuries, eccentric exercises, tendinopathy, laser therapy

## Abstract

Background

Achilles tendinopathy is a common overuse tendon injury, affecting athletes in running and similar sports. Repetitive overload of the Achilles tendon is the primary cause of inflammation, collagen degeneration, and tendon thickening. This study aims to investigate the efficacy of combining low-level laser therapy (LLLT) with eccentric exercises in treating midportion Achilles tendinopathy.

Methods

This prospective randomized controlled trial was conducted at the Sports Injury Centre, Vardhman Mahavir Medical College and Safdarjung Hospital, New Delhi, from 2019 to 2022. Sixty clinically diagnosed patients with midportion Achilles tendinopathy, aged 18 to 60, were randomly assigned to two groups: Group A received eccentric exercises with LLLT, and Group B received eccentric exercises with placebo LLLT. The Victorian Institute of Sport Assessment-Achilles (VISA-A) score and the visual analog scale (VAS) score were used to measure treatment effectiveness at baseline and three, six, 12, and 24 weeks.

Results

The study included 60 participants, with no dropouts observed. The mean age was 33.9 ± 8.3 years in Group A and 33.40 ± 8.64 years in Group B, with no significant difference between the groups (p = 0.821). Both groups showed significant improvement in VISA-A and VAS scores over time (p < 0.001), but there was no statistically significant difference between the groups at any time point (p > 0.05).

Conclusion

Adding LLLT to eccentric exercises did not provide significant additional benefits compared to eccentric exercises alone in treating midportion Achilles tendinopathy. Practitioners should prioritize evidence-based interventions, such as eccentric exercises, as the primary treatment modality while considering alternative therapies for adjunctive purposes. Further research is needed to explore additional modalities or combination therapies that may enhance outcomes for patients with Achilles tendinopathy.

## Introduction

Achilles tendinopathy is a common overuse tendon injury that can disable athletes involved in running sports. Excessive training intensity and duration can increase the incidence of such overuse injuries. Repetitive overload of the Achilles tendon is the primary cause of its tendinopathy and is commonly seen in athletes participating in sports, including racquet sports, volleyball, and soccer [1].

The primary treatment is conservative management, which includes cessation of sports activities, rest, ice compression, nonsteroidal anti-inflammatory drugs, gentle stretching, and eccentric strengthening [2-4]. Short-term effects of eccentric exercise include increased intensity changes in magnetic resonance imaging (MRI) and ultrasound (US). However, long-term effects include a reduction in the size and volume of the edematous Achilles tendon and a reduction in neovascularization in the area [4,5].

Laser therapy involves the local application of monochromatic light to the area of tenderness [6]. A study examining the anti-inflammatory effect of low-level laser therapy (LLLT) demonstrated a decrease in signal intensity on MRI and US, indicating a reduction of inflammation [7]. While Achilles tendinopathy has been extensively studied, the existing body of research examining the combined impact of laser therapy and eccentric strengthening has yielded varied outcomes, lacking consistent consensus. Therefore, we conducted this study to investigate the clinical effectiveness of LLLT combined with eccentric exercise in the treatment of midportion Achilles tendinopathy.

## Materials and methods

We conducted this prospective randomized controlled trial at the Sports Injury Centre, Vardhman Mahavir Medical College (VMMC) and Safdarjung Hospital, New Delhi, from 2019 to 2022, following ethical approval from the Institutional Ethics Committee of VMMC and Safdarjung Hospital (S. no. IEC/VMMC/SJH/Thesis/2019-10/69).

To determine our sample size, we referenced the study by Stergioulas et al., which reported that the pain intensity at 12 weeks was 33.0 ± 29.8 in the laser therapy group and 53.0 ± 19.5 in the placebo laser group [8]. Using these values, we calculated the minimum required sample size with 80% power and a 5% level of significance to be 25 patients in each group. Considering a potential loss to follow-up of 15%, the total sample size was increased to 60 patients, with 30 patients per group. Therefore, we included 60 patients aged 18 to 60 years with pain and tenderness localized at an area 2 cm to 6 cm proximal to the insertion of the Achilles tendon at the calcaneum. We excluded patients with comorbid conditions that could confound treatment or anticipated recovery, such as diabetes, hyperuricemia, hypercholesterolemia, neurological deficits, or systemic inflammatory diseases like rheumatoid arthritis or ankylosing spondylitis. Additionally, patients who had received prior treatments for Achilles tendinopathy within three months, including medications, physical therapy, corticosteroid injections, or any sclerosing therapy, were also excluded [9]. Patients with contraindications to LLLT, such as local site infections, dermatitis, eczema, or wounds, were also excluded [10].

All participants provided informed consent at the beginning of the study, and their rights were protected. All participants were assured of their confidentiality and were informed of their right to withdraw from the study at any point without any consequences. Patients meeting the study criteria were randomized using the block randomization method with sealed envelopes into two groups of 30 patients each. Group A received eccentric exercises with LLLT, and Group B received eccentric exercises with placebo LLLT. The treatment groups were masked to the patients.

Both groups were instructed by a qualified physiotherapist on a home-based exercise regimen. This regimen involved standing with both feet close to the edge of a step, with the heels hanging off the step, and using the uninvolved leg to lift onto the forefoot. The heel of the uninvolved foot was then slowly removed so the entire body weight fell on the involved leg. The plantar flexor muscle-tendon unit was eccentrically loaded by performing a heel drop until it was well behind and below the edge of the step, moving the ankle from plantar flexion to dorsiflexion. The uninvolved foot was used again to return to the start position, eliminating the concentric component from the injured leg. The exercise consisted of three sets of 15 repetitions, with both knees extended (for the gastrocnemius) and flexed (for the soleus), performed twice daily for 12 weeks, according to the modified Alfredson protocol for Achilles tendinopathy [11].

In Group A, LLLT was administered along with eccentric exercise. The physiotherapist administered laser therapy, targeting the localized tender areas with a laser probe of 820 nm wavelength. The probe consisted of a grid with 10 spots, each spot size 0.5 cm², emitting 4 J of energy per minute to an area of 5 cm² for two minutes, giving a total energy of 8 J per session. In Group B, placebo LLLT was given for two minutes using a dummy red light shining out of the end of the laser probe, visible on the skin.

The laser and placebo laser application methods were identical in both groups. Patients lay prone on a couch, and a trained physiotherapist delivered laser therapy over the Achilles tendon at the site of tenderness using the contact method. All patients in this group received two treatments per week for the first four weeks and one session per week for the next four weeks. Patients were instructed to follow the eccentric exercise regimen along with laser therapy.

The Victorian Institute of Sport Assessment-Achilles (VISA-A) score and the visual analog scale (VAS) score were used to measure treatment effectiveness at baseline and at three, six, 12, and 24 weeks following randomization in both groups. The VISA-A score is a valid, reliable, and specific questionnaire for Achilles tendon pathologies [12]. It assesses pain, function, and activity, scoring from 0 to 100, where 100 represents a healthy person and 0 represents the worst outcome. The VAS is a widely used, valid score [13] that assesses pain and quality of life on a scale of 0-100, where 0 represents "no pain at all" and 100 represents the worst pain imaginable.

Statistical analysis

Data were entered into MS Excel (Microsoft Corporation, Redmond, Washington, United States), and analysis was performed using IBM SPSS Statistics for Windows, Version 21.0 (Released 2012; IBM Corp., Armonk, New York, United States). Categorical variables were presented as numbers and percentages (%), and continuous variables were presented as mean ± standard deviation (SD) and median. The normality of data was tested using the Kolmogorov-Smirnov test. As the data were not normally distributed, non-parametric tests were used for statistical inference. The Wilcoxon-Mann-Whitney test was used to compare the two groups in terms of VISA-A and VAS at each timepoint. The Friedman test was used to explore changes in VISA-A and VAS over time within each group. Qualitative variables were compared using the Chi-square test or Fisher’s exact test, and we considered p < 0.05 statistically significant.

## Results

The study included 60 participants. No dropouts were observed throughout the study duration. The mean (± SD) age was 33.9 ± 8.3 years in Group A and 33.4 ± 8.6 years in Group B, with no significant difference between the groups (p = 0.821). Table 1 shows the number and percentage of patients in both groups in relation to age category, gender, and side of Achilles tendinopathy. There was no significant difference between the groups in terms of age. In Group A, 66.7% were male and 33.3% were female, while in Group B, 60.0% were male and 40.0% were female, indicating no significant difference in gender distribution. Additionally, the groups had no significant difference regarding the affected side (left foot: 53.3% in Group A vs. 56.7% in Group B; p = 0.795).

**Table 1:** Sample characteristics of the study population LLLT: Low-level laser therapy **Significant at p < 0.05. ^1^Fischer’s exact test. ^2^Chi-square test

Parameters		N (%)		p-value**
		Group A LLLT (n = 30)	Group B placebo (n = 30)	
Age (years)	21-30	13 (43.3%)	14 (46.7%)	0.942^1^
	31-40	11 (36.7%)	9 (30.0%)	
	41-50	5 (16.7%)	6 (20.0%)	
	51-60	1 (3.3%)	1 (3.3%)	
Gender	Male	20 (66.7%)	18 (60.0%)	0.592^2^
	Female	10 (33.3%)	12 (40.0%)	
Side	Right	14 (46.7%)	13 (43.3%)	0.795^2^
	Left	16 (53.3%)	17 (56.7%)	

**Figure 1:**
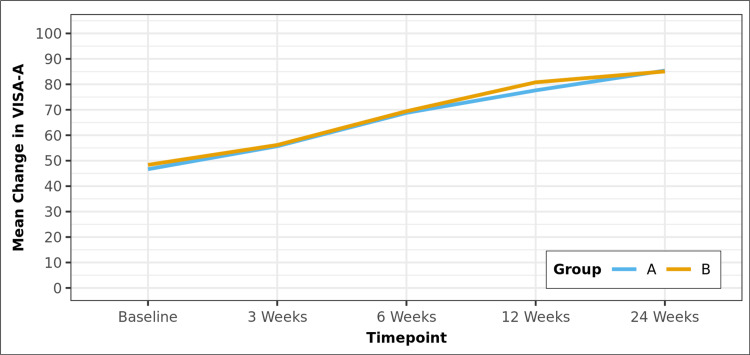
Line diagram depicting the change in VISA-A scores over time in the two groups VISA-A: Victorian Institute of Sport Assessment-Achilles

As shown in Table 2, the mean VISA-A score at baseline were similar in Groups A and B: 46.7 in Group A and 48.37 in Group B. At subsequent visits, both groups significantly improved their VISA-A scores (p < 0.001). The mean VISA-A scores at three, six, 12, and 24 weeks were 55.7, 68.8, 77.63, and 85.40 in Group A and 56.17, 69.43, 80.80, and 85.07 in Group B, respectively. There was no statistically significant difference between the groups at any timepoint (p > 0.05), as shown in the line diagram in Figure 1.

**Table 2:** Comparison of the test and the placebo groups change in VISA-A scores over time VISA-A: Victorian Institute of Sport Assessment-Achilles; NA, not applicable

Assessment time	VISA-A (mean ± SD)		p-value at each timepoint (Wilcoxon-Mann-Whitney test)
	Group A LLLT (n = 30)	Group B Placebo (n = 30)	
Baseline	46.67 ± 17.85	48.37 ± 19.11	0.636
Three weeks	55.70 ± 18.20	56.17 ± 19.24	0.976
Six weeks	68.80 ± 15.47	69.43 ± 17.11	0.888
12 weeks	77.63 ± 10.79	80.80 ± 10.22	0.328
24 weeks	85.40 ± 9.37	85.07 ± 9.46	0.750
p-value: change in VISA-A within each group (Friedman test)	<0.001	<0.001	NA
Overall p-value: change in VISA-A over time between groups (generalized estimating equations)	0.294		

**Figure 2:**
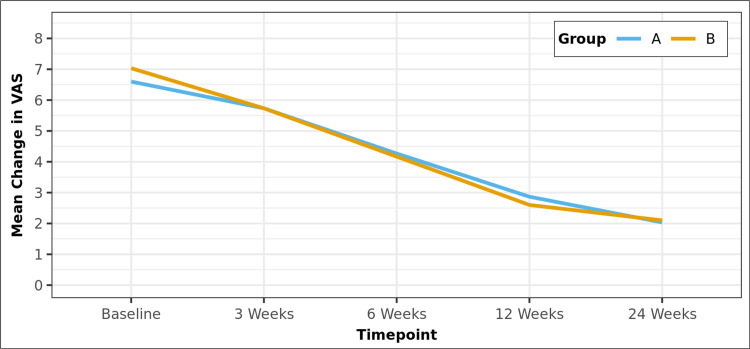
Line diagram depicting the change in VAS scores over time in the two groups VAS: Visual analog scale

Similarly, the mean VAS scores, as shown in Table 3, were similar in both Groups A and B at baseline (6.60 and 7.03, respectively; p = 0.197). Over time, both groups significantly improved their VAS scores (p < 0.001). The mean VAS scores at three, six, 12, and 24 weeks were 5.73, 4.27, 2.87, and 2.03 in Group A and 5.73, 4.17, 2.60, and 2.10 in Group B, respectively. There was no statistically significant difference between the groups at any timepoint (p > 0.05), as shown in the line diagram in Figure 2.

**Table 3:** Comparison of the test and placebo groups change in VAS scores over time VAS: Visual analog scale; NA, not applicable

Assessment time	VAS (mean ± SD)		p-value at each timepoint (Wilcoxon-Mann-Whitney test)
	Group A LLLT (n = 30)	Group B Placebo (n = 30)	
Baseline	6.60 ± 1.33	7.03 ± 1.52	0.197
Three weeks	5.73 ± 1.89	5.73 ± 1.96	0.976
Six weeks	4.27 ± 1.66	4.17 ± 2.09	0.707
12 Weeks	2.87 ± 1.41	2.60 ± 1.54	0.295
24 Weeks	2.03 ± 1.30	2.10 ± 1.27	0.724
p-value: change in VAS within each group (Friedman test)	<0.001	<0.001	NA
Overall p-value: change in VAS over time between groups (generalized estimating equations)	0.420		

## Discussion

This study analyzed the comparative effect of LLLT and eccentric exercise versus placebo LLLT and eccentric exercise on midportion Achilles tendinopathy. LLLT promotes angiogenesis and increased fibroblast activity, enhancing collagen production and remodeling. Eccentric exercise helps restore elasticity by significantly enhancing collagen synthesis [2,14].

The Achilles tendon is formed by the merging of the tendons from the gastrocnemius and soleus muscles, which attach to the calcaneus. It is encased in a non-true synovial sheath called the paratenon. The tendon receives its nutrition from the paratenon [15] and the interosseous vessels of the calcaneus in the distal part of the tendon, while a few intermuscular branches supply the proximal part. This leaves a relatively avascular area of 2 cm to 6 cm in the midportion of the tendon, making it more susceptible to degeneration following an injury or overuse. The matrix of a healthy tendon consists of an extracellular matrix primarily composed of collagen fibers embedded with tenocytes and tenoblasts, along with 5% fibroblast cells, endothelial cells, and smooth muscle cells [16].

The main role of the Achilles tendon is to enable plantar flexion at the ankle joint and assist in absorbing the body weight load caused by the external ground reaction force during running. Achilles tendinopathy is associated with repetitive microtrauma. The disease continuum starts with a reactive, noninflammatory proliferative response in the cell matrix of the Achilles tendon due to repetitive microtrauma. This repetitive microtrauma leads to inflammation of the tendon sheath. Insufficient time to recover from microtrauma results in the replacement of the matrix with excessive fibrin and adhesion of the tendon, causing tendinopathy [17-19]. Microscopically, there is noninflammatory collagen degeneration, fiber disorientation, hypercellularity, and neovascularization [20]. Cardinal symptoms include morning pain and ankle stiffness, with pain being the primary symptom that aggravates at the beginning and end of training. Clinical examination shows tenderness 2 cm to 6 cm proximal to tendon insertion with thickening of the fibers [21].

We conducted this study due to the limited evidence regarding the effectiveness of LLLT combined with eccentric exercises in managing Achilles tendinopathy. We hypothesized that combining laser treatment with eccentric exercise would result in faster and better recovery from Achilles tendinopathy.

Nogueira et al. conducted a systematic review to observe the effect of laser therapy on Achilles tendinopathy [6]. This review included 10 articles from PubMed and six from Capes Journal, of which three were used to review the outcomes [7,8,22].

Tumilty et al. used laser therapy with eccentric exercise for 12 weeks and measured outcomes using the VISA-A score. The study reported significant improvement in the laser therapy group, but the conclusion regarding effectiveness of LLLT cannot be drawn due to the low statistical power of their pilot study [22]. Stergioulas et al. used 12 laser therapy sessions with eccentric exercise for eight weeks and measured outcomes using the VAS score for pain and morning stiffness. The study concluded that there was accelerated recovery when laser therapy was combined with eccentric exercise [8]. Bjordal et al. used a single session of laser therapy for Achilles tendinopathy and concluded that inflammation was reduced following laser therapy [7].

Nogueira et al. concluded that there was clinical improvement after parallel application of laser therapy with eccentric exercise despite different parameters such as time, power, wavelength, and frequency of LLLT [6]. Similarly, a 2010 study by Tumilty et al. assessed the clinical effectiveness of LLLT in treating tendinopathy, including Achilles tendinopathy, through a systematic review and meta-analysis. This study included 25 controlled clinical trials and concluded that LLLT is potentially effective in treating tendinopathy [23].

In contrast, a 2012 Tumilty et al. randomized controlled trial found no significant difference between the group receiving eccentric exercise alone and the group receiving both eccentric exercise and laser therapy. Baseline VISA-A and VAS scores showed no difference between the two groups [9]. The VISA-A score at four weeks significantly favored the placebo group; however, no significant difference was observed at any other timepoint. The study concluded that LLLT did not demonstrate clinical effectiveness for Achilles tendinopathy [9]. Similarly, our study observed no significant difference in outcomes measured using VISA-A and VAS scores.

A recent systematic review and meta-analysis by Martimbianco et al., which included four trials, found no significant difference between the group receiving LLLT with eccentric exercise and the group receiving sham LLLT with eccentric exercises [24]. In our study, there was no significant difference between the mean VISA-A and mean VAS scores of both groups throughout the study. Therefore, our study does not support the effectiveness of LLLT combined with eccentric exercises over eccentric exercises alone.

The results of our study are consistent with those of Tumilty et al. (2012) and Martimbianco et al. [9,24]. Due to methodological similarities, our study is more comparable to Tumilty et al.’s 2012 study. However, direct comparison is not possible due to differences in the duration of LLLT application and follow-up periods. In our study, the tendon was irradiated at 10 points with 8 J of energy per session using an 820 nm probe compared to six points with 3 J per point using an 810 nm probe three times per week in Tumilty et al.’s study. Due to these conflicting findings, the evidence for the effectiveness of LLLT in treatment remains inconclusive.

Limitations

This study has several limitations. First, the follow-up period was limited to six months, which may not be sufficient to observe the long-term effects of LLLT and eccentric exercises on Achilles tendinopathy. This short follow-up period was based on previous research in similar contexts, which used follow-up periods ranging from three to six months [7,8,22]. This approach was chosen to avoid higher dropout rates that could compromise the validity of the study and to provide timely results, as our study was conducted as a postgraduate thesis. The sample size was small and nonspecific, lacking distinction between the general population and athletes, which may affect the generalizability of the results. Removing athletes from the study would have significantly reduced the number of participants recruited within the study's time frame, impacting the study's feasibility. Another limitation of this study is the absence of radiological investigations, such as MRI or US, in the diagnosis and assessment of Achilles tendinopathy. While clinical symptoms and physical examination were used as the basis for patient selection and evaluation, the inclusion of radiological imaging could have provided more detailed insights into the structural changes of the Achilles tendon. Also, the study did not account for variations in patients’ adherence to the home-based exercise regimen, which could influence the outcomes. We did not control for other potential confounding variables such as patients’ activity levels, diet, and use of other treatments or medications during the study period. The placebo effect in Group B (placebo LLLT) might have influenced the participants’ perception of pain and recovery, potentially affecting the results. Lastly, the variability in the application of LLLT, including differences in time, power, wavelength, and frequency, limits the ability to standardize the treatment protocol and compare results across studies.

## Conclusions

Our study aimed to investigate the effectiveness of combining LLLT with eccentric exercises in treating midportion Achilles tendinopathy. The findings revealed that adding LLLT to eccentric exercises did not provide significant additional benefits compared to eccentric exercises alone. LLLT does not offer a substantial advantage when used alongside eccentric exercises for midportion Achilles tendinopathy. Practitioners and clinicians should prioritize evidence-based interventions, such as eccentric exercises, as the primary treatment modality for Achilles tendinopathy while considering alternative therapies for adjunctive or complementary purposes. Further research is warranted to explore additional modalities or combination therapies that may enhance outcomes for patients with Achilles tendinopathy.

## References

[1] Selvanetti ACM, Puddu G (1997). Overuse tendon injuries: basic science and classification. Oper Tech Sports Med.

[2] Wilson F, Walshe M, O'Dwyer T, Bennett K, Mockler D, Bleakley C (2018). Exercise, orthoses and splinting for treating Achilles tendinopathy: a systematic review with meta-analysis. Br J Sports Med.

[3] van der Vlist AC, Winters M, Weir A (2021). Which treatment is most effective for patients with Achilles tendinopathy? A living systematic review with network meta-analysis of 29 randomised controlled trials. Br J Sports Med.

[4] Fyfe I, Stanish WD (1992). The use of eccentric training and stretching in the treatment and prevention of tendon injuries. Clin Sports Med.

[5] Mafi N, Lorentzon R, Alfredson H (2001). Superior short-term results with eccentric calf muscle training compared to concentric training in a randomized prospective multicenter study on patients with chronic Achilles tendinosis. Knee Surg Sports Traumatol Arthrosc.

[6] Nogueira AC Jr, Júnior Mde J (2015). The effects of laser treatment in tendinopathy: a systematic review. Acta Ortop Bras.

[7] Bjordal JM, Lopes-Martins RA, Iversen VV (2006). A randomised, placebo controlled trial of low level laser therapy for activated Achilles tendinitis with microdialysis measurement of peritendinous prostaglandin E2 concentrations. Br J Sports Med.

[8] Stergioulas A, Stergioula M, Aarskog R, Lopes-Martins RA, Bjordal JM (2008). Effects of low-level laser therapy and eccentric exercises in the treatment of recreational athletes with chronic achilles tendinopathy. Am J Sports Med.

[9] Tumilty S, McDonough S, Hurley DA, Baxter GD (2012). Clinical effectiveness of low-level laser therapy as an adjunct to eccentric exercise for the treatment of Achilles' tendinopathy: a randomized controlled trial. Arch Phys Med Rehabil.

[10] (2001). Electrotherapy: Evidence Based Practice. https://kclpure.kcl.ac.uk/portal/en/publications/electrotherapy-evidence-based-practice/publications/.

[11] Alfredson H, Pietilä T, Jonsson P, Lorentzon R (1998). Heavy-load eccentric calf muscle training for the treatment of chronic Achilles tendinosis. Am J Sports Med.

[12] Robinson JM, Cook JL, Purdam C (2001). The VISA-A questionnaire: a valid and reliable index of the clinical severity of Achilles tendinopathy. Br J Sports Med.

[13] Sherman SA, Eisen S, Burwinkle TM, Varni JW (2006). The PedsQL present functioning visual analogue scales: preliminary reliability and validity. Health Qual Life Outcomes.

[14] Langberg H, Ellingsgaard H, Madsen T, Jansson J, Magnusson SP, Aagaard P, Kjaer M (2007). Eccentric rehabilitation exercise increases peritendinous type I collagen synthesis in humans with Achilles tendinosis. Scand J Med Sci Sports.

[15] Carr AJ, Norris SH (1989). The blood supply of the calcaneal tendon. J Bone Joint Surg Br.

[16] Kirkendall DT, Garrett WE (1997). Function and biomechanics of tendons. Scand J Med Sci Sports.

[17] Longo UG, Ronga M, Maffulli N (2018). Achilles tendinopathy. Sports Med Arthrosc Rev.

[18] James SL, Bates BT, Osternig LR (1978). Injuries to runners. Am J Sports Med.

[19] Leadbetter WB (1992). Cell-matrix response in tendon injury. Clin Sports Med.

[20] Khan KM, Cook JL, Bonar F, Harcourt P, Astrom M (1999). Histopathology of common tendinopathies: update and implications for clinical management. Sports Med.

[21] Leppilahti J, Orava S, Karpakka J, Takala T (1991). Overuse injuries of the Achilles tendon. Ann Chir Gynaecol.

[22] Tumilty S, Munn J, Abbott JH, McDonough S, Hurley DA, Baxter GD (2008). Laser therapy in the treatment of achilles tendinopathy: a pilot study. Photomed Laser Surg.

[23] Tumilty S, Munn J, McDonough S, Hurley DA, Basford JR, Baxter GD (2010). Low level laser treatment of tendinopathy: a systematic review with meta-analysis. Photomed Laser Surg.

[24] Martimbianco AL, Ferreira RE, Latorraca CO, Bussadori SK, Pacheco RL, Riera R (2020). Photobiomodulation with low-level laser therapy for treating Achilles tendinopathy: a systematic review and meta-analysis. Clin Rehabil.

